# Pitfalls of the S-ICD therapy: experiences from a large tertiary centre

**DOI:** 10.1007/s00392-020-01767-x

**Published:** 2020-11-01

**Authors:** Kevin Willy, Florian Reinke, Benjamin Rath, Christian Ellermann, Julian Wolfes, Nils Bögeholz, Julia Köbe, Lars Eckardt, Gerrit Frommeyer

**Affiliations:** Clinic for Cardiology II: Electrophysiology, University Hospital Münster, Albert-Schweitzer-Campus 1, 48149 Munster, Germany

**Keywords:** Subcutaneous ICD, S-ICD, Pitfalls, Troubleshooting, ICD, Lead dysfunction, Ventricular arrhythmia

## Abstract

**Aim:**

The subcutaneous ICD (S-ICD) has evolved to a potential first option for many patients who have to be protected from sudden cardiac death. Many trials have underlined a similar performance regarding its effectiveness in relation to transvenous ICDs and have shown the expected benefits concerning infective endocarditis and lead failure. However, there have also been problems due to the peculiarities of the device, such as oversensing and myopotentials. In this study, we present patients from a large tertiary centre suffering from complications with an S-ICD and propose possible solutions.

**Methods and results:**

All S-ICD patients who experienced complications related to the device (*n* = 40) of our large-scale single-centre S-ICD registry (*n* = 351 patients) were included in this study. Baseline characteristics, complications occurring and solutions to these problems were documented over a mean follow-up of 50 months. In most cases (*n* = 23), patients suffered from oversensing (18 cases with T wave or P wave oversensing, 5 due to myopotentials). Re-programming successfully prevented further oversensing episode in 13/23 patients. In 9 patients, generator or lead-related complications, mostly due to infectious reasons (5/9), occurred. Further problems consisted of ineffective shocks in one patient and need for antibradycardia stimulation in 2 patients and indication for CRT in 2 other patients. In total, the S-ICD had to be extracted in 10 patients. 7 of them received a tv-ICD subsequently, 3 patients refused re-implantation of any ICD. One other patient kept the ICD but had antitachycardic therapy deactivated due to inappropriate shocks for myopotential oversensing.

**Conclusion:**

The S-ICD is a valuable option for many patients for the prevention of sudden cardiac death. Nonetheless, certain problems are immanent to the S-ICD (limited re-programming options, size of the generator) and should be addressed in future generations of the S-ICD.

**Graphic abstract:**

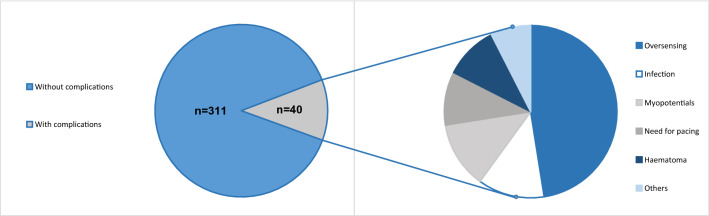

## Introduction

The subcutaneous ICD (S-ICD) (Boston Scientific, Natick, Massachusetts) is widely accepted as a valuable alternative to transvenous ICDs in a variety of clinical constellations requiring ICD therapy [1–5]. Due to positive experiences, the spectrum of indications has been constantly broadened so that the S-ICD has also been included in the current guidelines with a class IIa recommendation for the prevention of sudden cardiac death (SCD) [6]. In the light of the very recently published PRAETORIAN trial [7], which could show non-inferiority of the S-ICD compared to transvenous ICDs, a further upgrade in the next guideline revision for patients without indication for pacing is likely.

Despite these predominantly positive reports, typical complications already known from transvenous ICD systems may also occur with the S-ICD.

Oversensing due to an unfavourable ratio of signal (QRS complex) to noise (T-Wave or P-Wave) has been a rare complication of transvenous ICD systems as endocardial sensing and discrimination is often precise. In S-ICD systems correct discrimination is more difficult as a subcutaneous ECG is employed for signal detection. In contrast, oversensing in transvenous systems is most often caused by misinterpretation of supraventricular tachycardias, such as atrial fibrillation (AF) with rapid ventricular response (RVR) or lead dysfunctions, such as pace-sense lead fracture.

While detection algorithms of transvenous ICD systems have improved over the years and can be influenced by more sophisticated programming to facilitate correct differentiation of supraventricular and ventricular origin of the respective arrhythmia, programming features of the S-ICD are limited to changing of the sensing vectors (three possible options), changing of the therapy zones and modification of a gain factor of the QRS complex size.

Early studies, e.g. an analysis from the EFFORTLESS S-ICD registry, showed a rate of inappropriate shocks of 7% per year for the first generation S-ICD [8]. Inappropriate shocks were mainly attributed to T-wave-oversensing (39%) and supraventricular tachycardia above the discrimination zone (24%), which could be lowered by dual-zone programming and the addition of the SMART PASS filter to inappropriate shock rates of 3.5% per year [9].

A recently published analysis underlined an equivalent risk for inadequate therapy with transvenous and S-ICDs [10].

The other main concern about implantable cardiac devices is infection. A recent analysis by Viani et al. showed no re-infections in patients with lead extractions of transvenous ICDs and consecutive re-implantation of S-ICDs while 2/139 patients with re-implantation of transvenous ICDs had recurrent infection [11]. Data from a meta-analysis revealed a rate of pocket infections (2.7%) and delayed wound healing (0.6%) in S-ICD patients [12]. Finally, also the PRAETORIAN trial as the first randomized control trial could find no significant differences in overall complication rates with infectious complications occurring twice as often in transvenous than in S-ICDs (8 vs 4 patients).

## Materials and methods

The study was conducted in accordance with the guidelines of the Declaration of Helsinki. Between June 2010 and June 2020, a total of 351 S-ICD systems were implanted at our institution. In the present single-centre retrospective study, we enrolled all patients (*n* = 40, 11.4%) with complications related to the S-ICD. Indication for ICD implantation was in accordance to the current ESC guidelines. Patient characteristics are summarized in Table 1. Prior to implantation, S-ICD screening was performed with the automated screening tool. Patients were considered eligible for S-ICD™ implantation, if there was at least one suitable vector. All patients were scheduled for an intraoperative defibrillation test. In case of AF present at the time of scheduled operation, we performed transesophageal echocardiography for thrombus exclusion. In case of an unsuccessful test, the shock vector was changed to reversed polarity, the shock energy was raised or, if necessary, system components were repositioned intraoperatively using fluoroscopy. For follow-up, patients were examined at 6 weeks after implantation and every 3–6 months subsequently. Adverse events were documented during regular follow-up in 3–6 months’ intervals.

**Table 1:** Patient baseline characteristics

Baseline characteristics	Total (*n* = 40)
Male (*n*)	25 (63%)
Age (years)	40 ± 16.3
Left ventricular ejection fraction (%)	51.6 ± 12.9
Primary prevention (*n*)	21 (54%)
Previous transvenous ICD	2 (5%)
Underlying heart disease	
ICM	3 (7.5%)
DCM	4 (10%)
Electrical heart disease	8 (20%)
HCM	12 (30%)
Idiopathic VF	6 (15%)
Other (e.g. valvular)	7 (17.5%)

Data transformation and statistical analysis were performed using GraphPad PRISM 6.0 (San Diego, CA, USA) and the SPSS Statistics, version 20.0 (SPSS, Inc., Chicago, IL, USA). Continuous variables are presented as mean and standard deviation (SD), while categorical data are expressed as frequencies.

## Results

In total, we included 40 patients who presented undesirable effects of the S-ICD therapy. Of these patients, 64% were male and had a mean age of 40 years. The mean follow-up duration was about 4 years (see Table 1). Of note, there was no sudden cardiac death in the S-ICD cohort during follow-up raising suspicion of ventricular arrhythmia undersensing. All cardiac deaths were non-sudden and mostly due to heart failure.

Two patients had a history of transvenous ICD explantation. One was changed to the S-ICD after multiple electrode revisions due to oversensing and pocket infection. The other one suffered from inappropriate shock delivery due to T-wave-oversensing of the DDD-ICD. None of the patients with an infected S-ICD had a history of tv-ICD implantation.

**Fig. 1:**
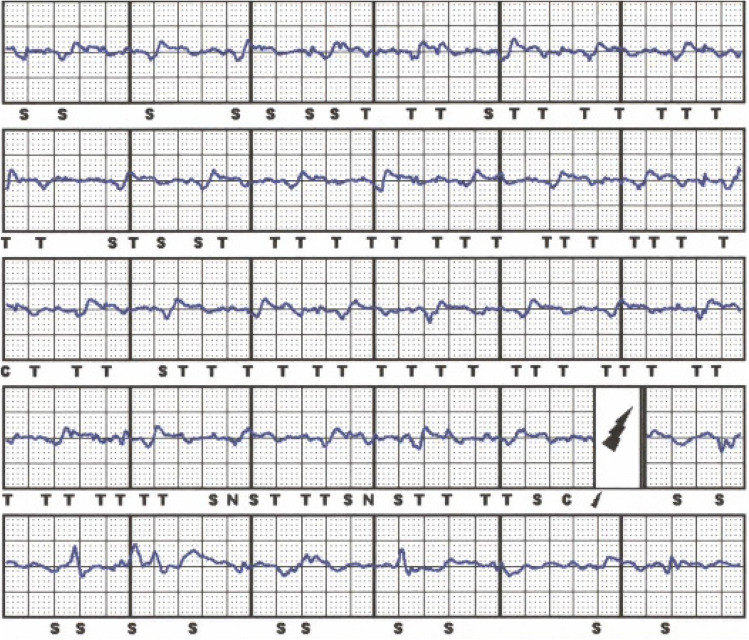
A patient with ischemic cardiomyopathy and sudden onset of T and also P wave oversensing resulting in an inappropriate shock. Because of the development of a left bundle branch block in course of the disease an indication for CRT emerged so that he received a CRT-ICD after these episodes

**Fig. 2:**
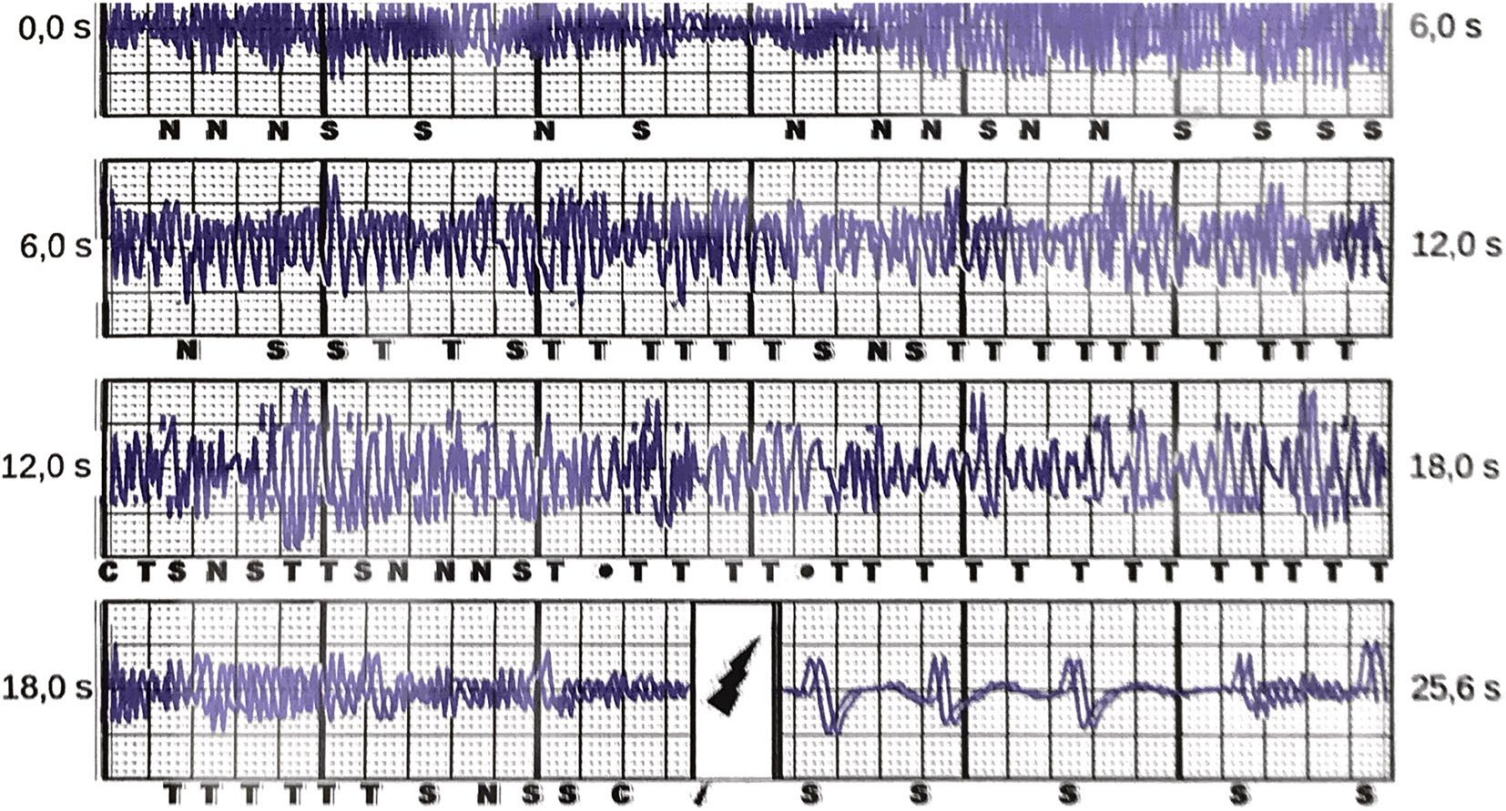
A young women who survived SCD due to VF was presenting in our emergency department after having experienced an S-ICD shock during yoga exercises. S-ICD interrogation revealed massive myopotential oversensing leading to tachycardia detection and a consecutive shock delivery. After muscular relaxation due to changing of the body position after IAS there was proper sensing of S-ECG signals again

23 of the 40 patients (58%) presented with oversensing. Table 2 Of these, 18 had T or P wave oversensing leading to inappropriate shock deliveries (IAS) in 15/18 patients (see Fig. 1). In the remaining 5 patients, oversensing was related to myopotentials resulting in IAS. 3 of these patients performed physical activity (yoga, sit-ups) and thereby induced oversensing episodes (see Fig. 2). In another patient with non-compaction cardiomyopathy, two inappropriate shock deliveries occurred due to AF with RVR and the ICD system was, therefore, changed to a transvenous system to enhance possibilities for discrimination of arrhythmia origin.

**Table 2:** Main results

Results	Total (*n* = 40)
Follow-up duration (months)	50.3 ± 41
Appropriate ICD therapy	5 (13%)
Complications due to	
Oversensing	19 (48%)
Myopotentials	5 (13%)
Need for pacing	2 (5%)
CRT indication	2 (5%)
Haematoma	4 (10%)
Hypermobility	2 (5%)
Infection	5 (13%)
IAS due to tachycardic AF	1 (2.5%)
Ineffective shocks	1 (2.5%)
Operative revisions	
Change to a transvenous ICD	8 (20%)
Keeping the S-ICD	4 (10%)
Explantation, no re-implantation	3 (8%)
Scheduled generator replacement	3 (8%)

In two patients, the S-ICD was explanted because of the need for antibradycardia stimulation. Both patients suffered from sick-sinus-syndrome so that we decided for DDD-ICDs for more physiological pacing and against an additional leadless pacer, especially as one patient also had monomorphic VT which could be accessible to antitachycardia pacing. Two further patients, of whom one patient also suffered from oversensing, underwent re-operation because of an emerging indication for CRT.

In a young patient with short-coupled variant of torsade de pointes ineffective shocks occurred during electrical storm despite effective ICD testing during implantation procedure.

**Fig. 3:**
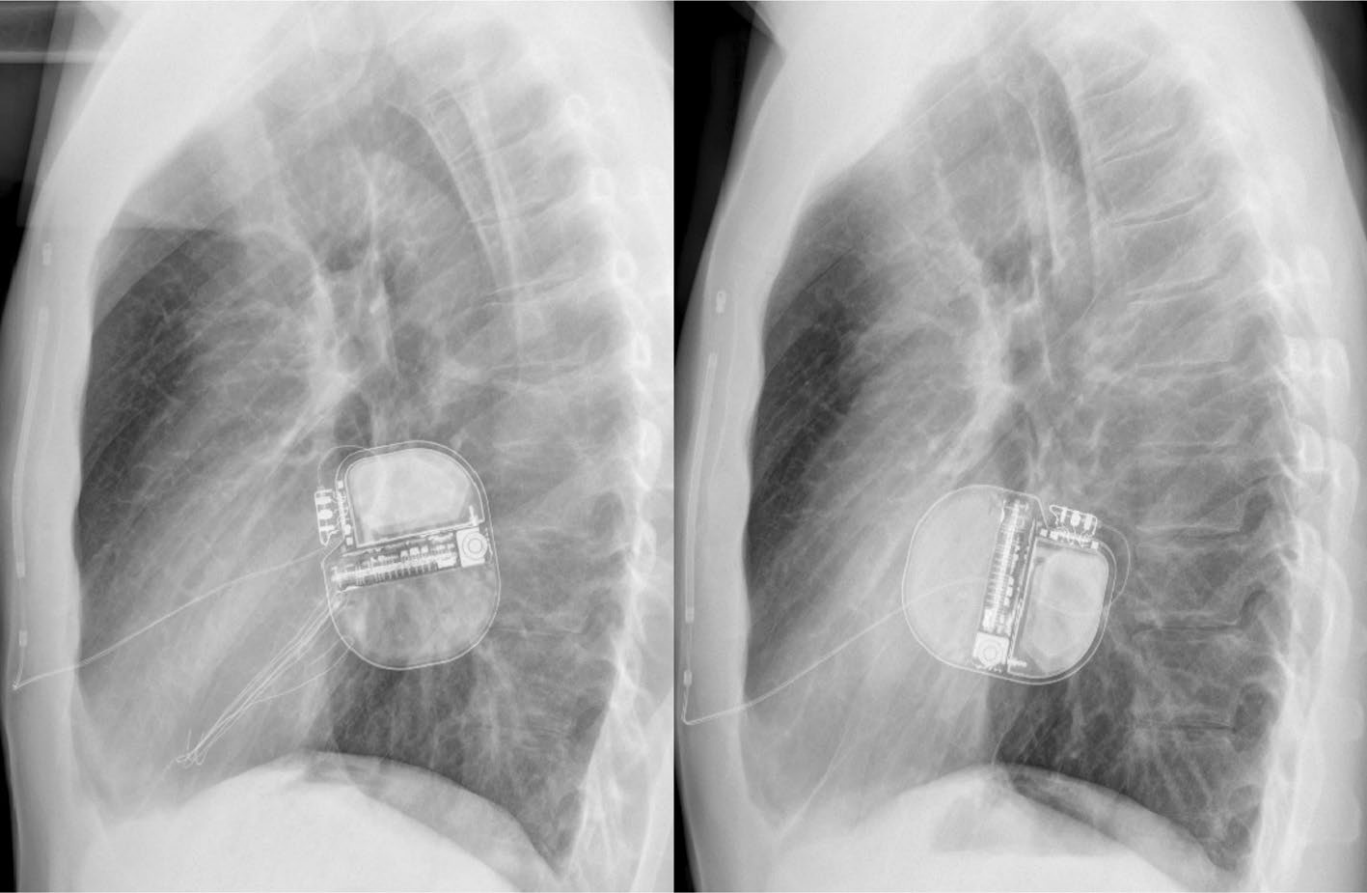
A patient with a recently implanted S-ICD which took about 90° dorsal rotation. The left picture showing X ray immediately postoperatively. The rotation, which led to discomfort and swelling of the pocket, is present in the right picture

Complications concerning the ICD pulse generator or lead were documented in 9 patients. 5 patients had infectious complications, of which 3 had to be re-operated. In 2 patients, the S-ICD had to be explanted due to perforation or purulent infection. In one patient, revision was necessary because of a mobile pulse generator (see Fig. 3). There were no thromboembolic complications concerning the defibrillation testing or during shock delivery during follow-up.

While in patients with T-wave-oversensing, re-programming was effective in preventing further episodes of oversensing, for example by changing of the sensing vector (13/18 patients), in all other types of complications revisions were necessary. In total, 14 patients were re-operated. In 7 patients, the S-ICD system was changed to a transvenous system, while 4 patients underwent revisions due to complications keeping the S-ICD as the active system at the end of the procedure. 3 patients refused re-implantation of any ICD device due to personal reasons. Another patient with arrhythmogenic right ventricular cardiomyopathy kept the S-ICD but had antitachycardia therapies switched off after multiple IAS due to oversensing. Transvenous ICD implantation was strongly recommended but was refused by the patient. Most complications occurred in patients with hypertrophic cardiomyopathy (HCM) (12) and electrical heart diseases/idiopathic ventricular fibrillation (8/4 resp.). For further information concerning the underlying diseases, please look at Table 1.

## Discussion

In the current study, we found S-ICD-related complications in 11.4% of patients in our large-scale S-ICD registry. Mostly, complications consisted of oversensing but also various other complications with, e.g. need for surgical revisions occurred.

In general, the development of the S-ICD has brought cardiologists and patients to a much more comfortable position. While before introduction of this device, all patients with an ICD indication had to undergo transvenous ICD implantation with its associated risks, the S-ICD now represents an attractive alternative option. Most young patients with ICD indication do not suffer from symptomatic heart failure or bradycardia but from cardiac diseases, which solely increase the risk for ventricular arrhythmia and SCD. Without a doubt, the vast majority of these patients, who receive a transvenous ICD will be subject to lead failure and will face operative revisions and implantation of additional leads throughout their lifetime. There is already evidence that lead-related complications are less common in S-ICD patients compared to patients with transvenous ICDs even during a relatively short follow-up according to a meta-analysis of several case–control studies [13], which is surprising as typical transvenous ICD complications, such as lead fracture, are mostly time-dependent. This is why especially in these patients, the S-ICD has become a popular first option for the prevention of SCD. This has been underlined very lately by the results of the PRAETORIAN trial which has shown non-inferiority of the S-ICD compared to transvenous ICDs in terms of safety and efficacy although numerically, there has been a trend to lower complication rates in the S-ICD but lower mortality in the transvenous ICD group [7]. However, there are also problems coming along with the S-ICD mainly being caused by its spare programming options. Noel et al. recently presented a case series of patients, in whom the S-ICD had to be explanted due to refractory oversensing issues [14]. In larger cohorts comparable to the size of the present study, the rate of oversensing was about 5–6% and might also depend on the sensing vector chosen [15, 16]. Also results from the Food and Drug Administration’s Manufacturer and User Facility Device Experience (MAUDE) database underlined that oversensing and infection are the prevailing problems of the S-ICD as they account for 2/3 (1604 complications reported in total) of all recorded complications in a large cohort of about 15.000 estimated S-ICDs [17]. In our cohort, only 3 of the 18 patients concerned underwent extraction of the S-ICD and implantation of a transvenous ICD reflecting the possibilities of re-programming, e.g. also with manual vector setup, in terms of preventing further oversensing episodes. In all of the 15 patients who kept the S-ICD despite documented oversensing, further oversensing episodes could be completely prevented by such measures. Furthermore, also lead or pulse generator repositioning could be discussed before changing to a transvenous system but was not performed in our study. Only in one patient, repositioning was necessary and caused by pulse generator rotation leading to patient discomfort (s. Fig. 3). However, reprogramming options are still very limited compared to transvenous ICDs and detection and therapy algorithms of the device can still not be influenced by the physician. Surely, measures, such as extension of the detection time intervals or direct influence on the discrimination criteria, would be helpful upgrades of the device. Furthermore, advancements of the subcutaneous ECG or a loop recorder function will further expand diagnostic and therapeutic features of the S-ICD.

In case of system infection, contralateral re-implantation as it would be performed in transvenous ICDs is not possible due to the decisive role of generator placement at the left hemithorax on S-ICD function. As a solution, patients either have to be bridged with a wearable defibrillator until re-implantation after completion of the antibiotic treatment or the system has to be changed to a transvenous ICD. The larger size of the pulse generator and the more exposed position might predispose to more pocket complications as discussed earlier. In our cohort, risk of infectious complications was 1.3% and well below the risk determined in a recent meta-analysis [12]. Three more patients suffered from pocket hematoma and had to be revised for this reason. Two of these had compromised blood coagulation (dual antiplatelet therapy in one patient, liver cirrhosis in the other). None of these pocket hematomas led to explantation of the S-ICD. Nonetheless, the bigger size of the S-ICD generator should be subject to future system advancement as it might contribute to hemorrhagic and infectious complications and might increase patient acceptance and device comfort.

Concerning the influence of the underlying cardiac disease, it has to be underlined that complications were quite prevalent in patients with HCM which has extensively proven in literature before [3, 18, 19]. However, there were also 14 patients with complications with idiopathic VF or electrical heart disease as well as 3 patients with ischemic and 4 patients with dilated cardiomyopathy. This illustrates that the influence of the underlying heart disease is not as severe as one might presume.

An ineffective shock was only observed in a single patient, in whom after many effective shocks, one episode of VF could be terminated only with the fourth shock. Due to a high overall arrhythmia burden without convincing options of antiarrhythmic treatment (short-coupled variant of torsade-de-points), decision was made for S-ICD extraction and implantation of transvenous ICD to establish the opportunity for overdrive pacing and administering of bradycardic drugs, such as verapamil, which has to be shown to be helpful in this rare entity [20].

## Limitations

This study has many limitations mainly caused by its retrospective nature. Furthermore, it has to be underlined that follow-up was unstructured and, therefore, not equally long for all patients. Patients were not scheduled for further investigations at our institution if they preferred an outpatient aftercare closer to their homes. However, we regularly received information from the outpatient cardiologists if problems occurred.

## Conclusion

All in all, the S-ICD is a valuable option in many patients for the prevention of SCD. However, decision for the right ICD system should be discussed carefully with each patient individually, so that the data presented in the manuscript might help presenting advantages but also disadvantages of the S-ICD more properly. There are no heart diseases that are especially prone for problems with the S-ICD, although S-ICD therapy is still difficult in patients with HCM and bundle branch blocks that increase the risk for dysfunction and inappropriate sensing. Most oversensing episodes can be prevented by reprogramming and patient education (prevention of myopotentials). Infectious complications are rare but often require extraction of the system.
